# Association Between Increased Levels of Cystatin C and the Development of Cardiovascular Events or Mortality: A Systematic Review and Meta-Analysis

**DOI:** 10.5935/abc.20180171

**Published:** 2018-12

**Authors:** Caroline Fuchs Einwoegerer, Caroline Pereira Domingueti

**Affiliations:** Universidade Federal de São João del-Rei, Divinópolis, MG - Brazil

**Keywords:** Cardiovascular Diseases/mortality, Cystatin C, Coronary Artery Disease, Myocardial Infarction, Renal Insufficiency, Chronic, Meta-Analysis as Topic

## Abstract

**Background:**

Cystatin C seems promising for evaluating the risk of cardiovascular events and mortality.

**Objective:**

To evaluate the association between high levels of cystatin C and the development of cardiovascular events or mortality.

**Methods:**

The articles were selected in the Medline/PubMed, Web of Science, and Scielo databases. The eligibility criteria were prospective cohort observational trials that assessed the association of high serum levels of cystatin C with the development of cardiovascular events or mortality in individuals with normal renal function. Only studies that evaluated the mortality outcome compared the fourth with the first quartile of cystatin C and performed multivariate Cox’s proportional hazard regression analysis were included in the meta-analysis. A p value < 0,05 was considered significant.

**Results:**

Among the 647 articles found, 12 were included in the systematic review and two in the meta-analysis. The risk of development of adverse outcomes was assessed by eight studies using the hazard ratio. Among them, six studies found an increased risk of cardiovascular events or mortality. The multivariate regression analysis was performed by six studies, and the risk of developing adverse outcomes remained significant after the analysis in four of these studies. The result of the meta-analysis [HR = 2.28 (1.70-3.05), p < 0.001] indicated that there is a significant association between high levels of cystatin C and the risk of mortality in individuals with normal renal function.

**Conclusion:**

There is a significant association between high levels of cystatin C and the development of cardiovascular events or mortality in individuals with normal renal function.

## Introduction

Cardiovascular diseases are the leading cause of death in the world, accounting for 31% of all deaths. In 2015, an estimated 17.7 million people died from cardiovascular diseases, mainly coronary heart disease, cerebrovascular disease, and peripheral arterial disease.^1^ In addition to high mortality, cardiovascular diseases are also associated with high morbidity, contributing to a significant share of public expenditure on health.^2^

Chronic kidney disease is an important risk factor for the development of cardiovascular events, and is also responsible for increased morbidity and mortality in patients with cardiovascular disease^3^. Cystatin C consists of a marker of renal dysfunction that has been shown to be more sensitive than serum creatinine to assess the early stages of renal failure^4^. It consists of a relatively stable cysteine protease inhibitor, produced in all nucleated cells at a constant rate.^5^

Because of the greater sensitivity of cystatin C for detecting the early and milder stages of renal dysfunction, the evaluation of serum levels has been shown to be promising for assessing the risk of cardiovascular events and mortality in individuals with apparently normal renal function. In recent years, some studies have demonstrated an association between serum cystatin C levels and the development of AMI.^6^ In addition, cystatin C has been shown to be useful for prognostic stratification in patients with ACS.^7^

However, there is a divergence between the results of studies performed to date on the clinical utility of cystatin C to assess the risk of cardiovascular events and mortality in individuals with normal renal function.^3,7,8^ Although some meta-analyses.^9-12^ have been published on the subject, the population of the studies selected did not consist only of patients with normal renal function. Therefore, the objective of this systematic review and meta-analysis was to evaluate the association between high levels of cystatin C and the development of cardiovascular events or mortality in subjects with normal renal function.

## Methods

This systematic review followed the recommendations of the Preferred Reporting Items for Systematic Reviews and Meta-analyses (PRISMA) statement.^13^

### Articles Selection

The articles selection was performed through the data bases *Medline (PubMed)* and *Web of Science*, using the descriptors “cystatin C”, “post-gamma-globulin”, “post-gamma globulin”, “neuroendocrine basic polypeptide”, “basic polypeptide, neuroendocrine”, “cystatin 3”, “gamma-trace”, “gamma trace”, combined with the descriptors “acute coronary syndrome”, “acute coronary syndromes”, “coronary syndrome, acute”, “coronary syndromes, acute”, “syndrome, acute coronary”, “syndromes, acute coronary”, “myocardial infarction”, “infarction, myocardial”, “infarctions, myocardial”, “myocardial infarctions”, “cardiovascular stroke”, “cardiovascular strokes”, “stroke, cardiovascular”, “strokes, cardiovascular”, “heart attack”, “heart attacks”, “myocardial infarct”, “infarct, myocardial”, “infarcts, myocardial”, “myocardial infarcts”, “myocardial ischemia”, “ischemia, myocardial”, “ischemias, myocardial”, “myocardial ischemias”, “ischemic heart disease”, “heart disease, ischemic”, “disease, ischemic heart”, “diseases, ischemic heart”, “heart diseases, ischemic”, “ischemic heart diseases”, using the connector “AND” between the terms. The Medical Subject Headings (MeSH) was used to define these descriptors.

The selection of the articles was also performed in *Scielo* database, using the descriptors “cystatin C” with the Boolean operators “acute coronary syndrome”, “coronary disease”, “coronary heart disease”, “myocardial infarction”, “heart attack”, “cardiac attack”, “myocardial ischemia”, “heart disease, ischemic”, “ischemia, myocardial” and “ischemic heart disease” using AND connector between the terms. The Descriptors in Health Sciences (DeCS) was used to define these descriptors.

### Eligibility criteria

The eligibility criteria were established according to the PRISMA recommendation,^13^ and consist of prospective cohort observational studies written in English, Portuguese or Spanish evaluating the association between high levels of cystatin C, and the development of cardiovascular events or mortality in individuals with normal renal function. There was no restriction of the period of publication of articles in the research. PECOS strategy was used to elaborate the research question:

1. Population of interest: Individuals with normal renal function.
2. Exposure: High levels of cystatin C.
3. Outcome: Cardiovascular events or mortality.
4. Study Design: Prospective cohort.

### Extracting data from selected articles

The following data were obtained from the studies that met the eligibility criteria: method used for measuring serum levels of cystatin C, patient group size, patient follow-up time, patient age range, criterion used to define normal renal function, outcome obtained in the study, outcome assessed, study population, patient classification, and parameters included in Cox proportional hazards multivariate regression analysis.

### Quality of the selected articles

The methodological quality evaluation process of the studies included in the review was carried out by two reviewers using the Newcastle-Ottawa Scale (NOS)^14^ questionnaire for cohort studies, which contains the following categories of evaluation: cohort selection; comparability of the cohort and outcome. The quality of the study is indicated with a maximum of nine stars, with only one star being allowed to be assigned in the selection and outcome categories, and two stars in the comparability category. The articles reaching a score of five to six stars were considered as articles of good methodological quality, and those with seven or more stars were considered articles of excellent methodological quality.

### Meta-Analysis

The meta-analysis included only those studies that assessed the outcome all-cause mortality comparing the fourth quartile of cystatin C with the first quartile and that conducted multivariate regression analysis of Cox proportional hazards. The *hazard ratio* value and the 95% confidence interval adjusted by the multivariate regression analysis were used in the meta-analysis and the I^2^ test was used to assess the heterogeneity among the studies. The studies were considered heterogeneous when I^2^ > 50% and p < 0.10. When there was homogeneity, the *hazard ratio* was calculated using the fixed effect model. The distribution of the studies included in the meta-analysis was analyzed by a funnel plot. The statistical software *Review Manager* version 5.3 was used to perform the statistical analysis. The p value < 0.05 was considered significant.

## Results

### Literature search

**Figure 1:**
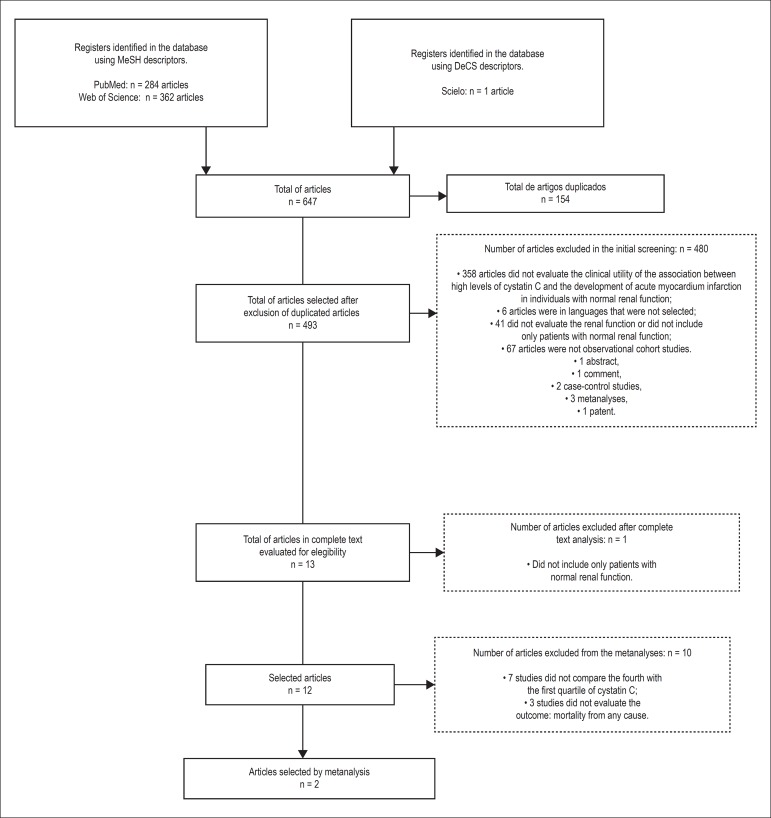
Flow chart of the articles selected for review, according to the elegibility criteria used in the study.

The initial search through the descriptors in the electronic databases resulted in a total of 647 articles. After completing the selection steps, 12 articles were included in the systematic review, and two were included in the meta-analysis. The flow chart for the selection of articles according to the eligibility criteria is presented in Figure 1.

### Characteristics and results of selected articles

The studies that met the eligibility criteria were published between 2007 and 2016 and their characteristics are found in Table 1.

**Table 1:** Characteristics of selected studies

Author/Year	Number of patients/ Age group	Study population	Patient follow-up time	Evaluated outcome
Sai et al., 2016^19^	277/64	Patients undergoing PCI	5 years and 3 months	Cardiovascular death, cerebrovascular death, ACS including non-fatal AMI and unstable angina, non-fatal stroke and hospitalization due to worsening CHF
Bansal et al., 2016^15^	2410/40,2 ± 3,6	Patients at risk for cardiovascular events who underwent echocardiography	10 years	Left ventricular hypertrophy
Abid et al., 2016^7^	127/58 ± 11,65	Patients with STEMI and NSTEMI	1 year	Cardiovascular death, myocardial reinfarction, NSTEMI, HF
Woitas et al., 2013^18^	2356/64 ± 10	Patients with CAD and healthy individuals	10 years	Cardiovascular death and death from any cause
Dupont et al., 2012^8^	615/65 ± 11	Patients with CHF who underwent coronary angiography	3 years	Death from any cause, non-fatal AMI and non-fatal stroke
Gao et al., 2011^21^	13 8/65,4 ± 11,0	Patients with chronic or new onset systolic CHF	3 years	Cardiovascular death, development or progression of HF requiring hospitalization, intravenous treatment of HF within the first 3 days after admission, cardiac transplantation
Keller et al., 2009^17^	1827/62	Patients with stable CAD or ACS	4 years	Cardiovascular death
Gao et al., 2009^22^	160/60	Patients with stable, unstable angina and AMI and healthy individuals	6 months	AMI, cardiovascular death, refractory angina, PCI and angiography
Alehagen et al., 2009^20^	464/65 to 87	Patients with CHF	10 years	Cardiovascular death
Acuna et al., 2009^16^	203/66,6 ± 13,16	Patients with STEMI and NSTEMI	1 years and 3 months	Cardiovascular death and HF
Koenig et al., 2007^24^	466 3/≥ 65	Elderly subjects (≥ 65 years)	9,3 years	Death from any cause, cardiovascular death, incident HF, stroke and AMI
Ix et al., 2007^23^	990/67	Patients with a history of AMI, angiographic evidence of stenosis greater than 50% in 1 or more coronary vessels, evidence of treadmill-induced ischemia or nuclear testing, or history of coronary artery bypass grafting	3 years and 1 month	Cardiovascular death, non-fatal AMI, stroke, death from all causes and HF

AMI: Acute Myocardial Infarction; HF: Heart failure; CHF: congestive heart failure; NSTEMI: Non-ST-segment elevation myocardial infarction; PCI: Percutaneous coronary intervention; ACS: acute coronary syndrome; STEMI: ST-segment elevation myocardial infarction; CAD: Coronary artery disease.

### Population

The population of the studies analyzed consisted of patients at risk for cardiovascular events,^15^ with ST-elevation myocardial infarction (STEMI), non-ST-segment elevation myocardial infarction (NSTEMI),^7,16^ and stable coronary artery disease (CAD),^17,18^ ACS,^17^ patients undergoing percutaneous coronary intervention,^19^ with congestive heart failure (CHF),^20,21^ with CHF who underwent coronary angiography,^9^ with stable angina and AMI,^22^ with a history of AMI that had angiographic evidence of stenosis greater than 50%,^23^ or healthy elderly individuals (older than 65 years).^24^

### Sample size, age group and follow-up time

The sample size varied from 127 to 4,663 individuals, and the sample number of 25% (n = 3)^8,20,23^ of the studies ranged from 400 to 1000 individuals, 41.67% (n = 5)^7,16,19,21,22^ of the studies had a sample number of less than 300 patients, and 33.33% (n = 4)^15,17,18,24^ had a sample size greater than 1000. The mean age ranged from 37 to 87 years, with 41.66% (n =5)^7,8,16,18,21^ of the studies evaluating both adult and elderly population (over 60 years), 50% (n = 6)^17,19,20,22-24^ evaluating only the elderly population, and one study [8,33% (n = 1)]^15^ analyzing only the adult population (below 60 years). The study follow-up time ranged from 6 months to 10 years, with 25% (n = 3)^7,16,22^ accompanying patients for less than 15 months, 41.67% (n = 5)^8,17,19,21,23^ following for 3 to 6 years, and 33.33% (n = 4)^15,18,20,24^ following for a period of more than 9 years.

### Outcome

The main outcomes evaluated by the studies were cardiovascular death (n = 10; 83.33%),^7,16-24^ heart failure (n = 6; 50%),^7,16,19,21,23,24^ and acute myocardial infarction (n = 6; 50%),^7,8,19,22-24^ followed by stroke (n = 4; 33,33%),^8,19,23,24^ death from any cause (n = 3; 35%,)^8,23,24^ and unstable angina (n = 2; 16,67%).^19,22^ Only one study (8.33%) evaluated each of the following outcomes: cerebrovascular death,^19^ left ventricular hypertrophy,^15^ myocardial reinfarction,^7^ need for percutaneous coronary intervention,^22^ and angiography.^22^

### Method for dosing cystatin C and criteria for the definition of normal renal function

The cystatin C dosing method and the criteria used to define normal renal function in the selected studies are shown in Table 2. The methods used for cystatin C dosing were immunonephelometry [41.67% (n = 5)],^15-18,23^ immunoturbimetry [33.33% (n = 4)],^7,8,19,20^ and immunoenzymatic assay [8.33% (n = 1)].^22^ Two studies (16.66%)^21,24^ did not report the method used for cystatin C dosing. The criteria used to define normal renal function were the GFR, estimated by the MDRD equation, above 60 mL/min/1.73 m^2^ [66.67% (n = 8)],^7,8,16-19,23,24^ the GFR, estimated by the CKD-EPI equation based on cystatin C, above 60 mL/min/1.73 m^2^, and normal albuminuria [8,33% (n = 1)]^15^ and serum creatinine levels below 115 µmol/L [8,33% (n = 1)].^20^ Two studies (16.67%)^21,22^ did not mention the method of evaluation of renal function.

**Table 2:** Method of dosing cystatin C and criteria for the definition of normal renal function in the selected studies

Author/Year	Method of dosing cystatin C	Criteria used to define normal renal function
Sai et al., 2016^19^	Immunoturbimetry	GFR calculated using the MDRD equation > 60 mL/min/1.73m^2^
Bansal et al., 2016^15^	Immunonephelometry	GFR based on cystatin C using the equation CKD-EPI > 60 mL/min/1.73 m^2^ and normal albuminuria
Abid et al., 2016^7^	Immunoturbimetry	GFR calculated using the MDRD equation > 60 mL/min/1.73 m^2^
Woitas et al., 2013^18^	Immunonephelometry	GFR calculated using the MDRD equation > 60 mL/min/1.73 m^2^
Dupont et al., 2012^8^	Immunoturbimetry	GFR calculated using the MDRD equation > 60 mL/min/1.73 m^2^
Gao et al., 2011^21^	NI	NI
Keller et al., 2009^17^	Immunonephelometry	GFR calculated using the MDRD equation > 60 mL/min/1.73 m^2^
Gao et al., 2009^22^	Enzyme immunoassay	NI
Alehagen et al., 2009^20^	Immunoturbimetry	Creatinine < 115 µmol/L
Acuna et al., 2009^16^	Immunonephelometry	GFR calculated using the MDRD equation > 60 mL/min/1.73 m^2^
Koenig et al., 2007^24^	NI	GFR calculated using the MDRD equation > 60 mL/min/1.73 m^2^
Ix et al., 2007^23^	Immunonephelometry	GFR calculated using the MDRD equation > 60 mL/min/1.73 m^2^

MDRD: Modification of diet in renal disease; NI: Not informed; GFR: Glomerular filtration rate.

### Classification of patients and variables included in the multivariate regression analysis

The way patients were classified in each of the selected studies, and the variables included in the multivariate Cox proportional hazards regression analysis are presented in Table 3, while the results of the studies are presented in Table 4. Among the studies included in this systematic review, five (41.66%)^8,17,18,20,23^ classified patients according to cystatin C quartiles; three (25%)^8,21^ classified patients according to whether or not there were fatal or non-fatal cardiovascular events; two (16.66%)^19,21^ divided the patients according to the median of cystatin C; one study (8.33%)^17^ classified patients according to whether or not they developed cardiovascular death; another study (8.33%)^18^ compared patients with coronary disease in relation to the healthy control group; a study (8.33%)^22^ classified the patients into four groups: stable angina, unstable angina, AMI and healthy control group; another study (8.33%)^15^ classified patients according to the GFR estimated by the CKD-EPI equation based on cystatin C: between 60 and 75 mL/min/1.73 m^2^; between 76 and 90 mL/min/1.73 m^2^; and above 90 mL/min/1.73 m^2^; two other studies (16.66%)^7,16^ further divided patients into two groups according to cystatin C levels above or below 0.95 mg/L and above and below 1.2 mg/L; and one study^24^ divided them according to high or low levels of cystatin C without mentioning the cutoff point.

**Table 3:** Classification of patients and variables included in multivariate regression analysis of Cox proportional hazards in selected studies

Author/Year	Classification of patients	Variables included in the multivariate regression analysis
Sai et al., 2016^19^	Patients with cystatin C levels above (n = 138) and below (n = 139) median. (Median = 0.637)	BMI, hypertension, HbA1c, HDL, BNP, cystatin C.
Bansal et al., 2016^15^	GFR between 60 and 75 mL/min/1.73 m^2^ (n = 29).GFR between 76 and 90 mL/min/1.73m^2^ (n = 153).GFR > 90 mL/min/1.73 m^2^ (n = 2228).	Age, gender, race, smoking, DM, LDL, HDL, albuminuria, BMI, systolic blood pressure.
Abid et al., 2016^7^	Patients who developed fatal (n = 6) or non-fatal (n = 26) cardiovascular events and patients who did not develop these events.Patients with cystatin C levels> 1.2 mg/L and <1.2 mg/L	NA
Woitas et al., 2013^18^	Patients with coronary disease (n = 2,346) and control group (n = 652).First quartile < 0.8 mg/L (n = 731).Second quartile 0.81 to 0.91 mg/L (n=769).Third quartile 0.91 to 1.06 mg/L (n=752).Fourth quartile > 1.07 mg/L (n=746)	Hypertension, HDL, LDL, triglycerides, statin use, smoking, DM, usPCR, GFR CKD-EPI based on creatinine, age, gender, BMI
Dupont et al., 2012^8^	Cystatin C quartiles.	NA
Gao et al., 2011^21^	Patients who developed fatal or non-fatal (n = 21) cardiovascular events and patients who did not develop these events (n = 117).Patients with cystatin C levels above the median and below the median (0.9 mg/L).	Male gender, history of hypertension, high creatinine, reduced triglycerides, high homocysteine, high usPCR, high cystatin C.
Keller et al., 2009^17^	Patients with cardiovascular death (n = 66) and patients without cardiovascular death (n = 1761).Cystatin C quartiles.	Age, gender, BMI, smoking, DM, hypertension, LDL/HDL ratio, PCR, GNP.
Gao et al., 2009^22^	Patients with stable angina (n = 34), patients with unstable angina (n = 56), patients with AMI (n = 36) and control group (n = 34).Patients who developed fatal or non-fatal (n = 26) cardiovascular events and patients who did not develop these events (n = 22).	NA
Alehagen et al., 2009^20^	First quartile: < 1.22 mg/L (n = 109).Second quartile: 1.22 to 1.42 mg/L (n = 120).Third quartile: 1.43 to 1.66 mg/L (n = 117).Fourth quartile: > < 1.66 mg/L (n = 118).	NA
Acuna et al., 2009^16^	Patients with cystatin C levels> 0.95 mg/L (n = 63) and ≤ 0.95 mg/L (n = 76)	NA
Koenig et al., 2007^24^	Patients with high (n = 1261) and reduced levels of cystatin C (n = 1347)	NA
Ix et al., 2007^23^	First quartile: ≤ <0.91 mg/L (n = 239).Second quartile: 0.92 to 1.05 mg/L (n = 248).Third quartile: 1.06 to 1.29 mg/L (n = 262).Fourth quartile:> ≥ <1.30 mg/L (n = 241).	Age, gender, race, smoking, DM, hypertension, previous AMI, smoking, HDL, BMI, CRP.

DM: Diabetes mellitus; HDL-high density lipoprotein; AMI: Acute Myocardial Infarction; BMI: Body mass index; LDL: low density lipoprotein; NA: Not applicable; CRP: C-reactive protein; GFR: Glomerular filtration rate; usPCR: Ultra-sensitive C-reactive protein.

**Table 4:** Results of selected studies

Author/Year	Result
Sai et al., 2016^19^	Proportion of patients with cystatin C levels> 0.637 mg/L who developed fatal or non-fatal cardiovascular events was higher than in patients with cystatin C < 0.637 mg/L [22 (15.9%) x 7 (5, 0%), p = 0.0025].Risk of fatal or non - fatal cardiovascular events in patients with cystatin C levels > 0.637 mg/L was greater than in patients with cystatin levels < 0.637 mg/L [(univariate) HR = 1.37 (1.10 - 1.66), p = 0.004; HR (multivariate) = 1.30 (1.01 - 1.63), p = 0.0038].
Bansal et al., 2016^15^	Risk of left ventricle hypertrophy was higher in patients with GFR between 60 and 75 ml/min/1.73 m^2^ than in those with GFR > 90 ml/min/1.73 m^2^ [(univariate) HR = 10.12 (5.22 - 15.02), p < 0.001; HR (multivariate analysis) = 5.63 (0.90 - 10.36), p = 0.02]Risk of left ventricular hypertrophy was higher in patients with GFR between 76 and 90 mL/min/1.73m^2^ than in those with GFR> 90 mL/min/1.73 m^2^ [HR (univariate analysis) = 3.48 (1, 29 - 5.68), p = 0.002].
Abid et al., 2016^7^	Patients who developed non-fatal cardiovascular events showed higher levels of cystatin C compared to patients who did not develop these events (1.19 ± 0.4 mg/L x 1.01 ± 0.35 mg/L, p = 0.01)Patients who developed fatal cardiovascular events showed higher levels of cystatin C compared to patients who did not develop these events (1.21 ± 0.36 mg/L x 0.96 ± 0.27 mg/L, p = 0.03)Survival of patients with cystatin C levels < 1.2 mg/L was higher than in patients with cystatin levels > 1.2 mg/L (p < 0.01).
Woitas et al., 2013^18^	Patients with CAD showed higher levels of cystatin C than the control group (1.02 ± 0.44 mg/L x 0.92 ± 0.26 mg/L, p = 0.065Risk of cardiovascular death and death from any cause of fourth quartile patients was higher than that of first quartile patients [HR (univariate) = 4.82 (3.69 - 6.29), p < 0.001; HR (multivariate) = 2.05 (1.48 - 2.84), p < 0.001].Risk of cardiovascular death and death from any cause of third quartile patients was higher than that of first quartile patients [HR (univariate) = 2.11 (1.58 - 2.81), p < 0.001; HR (multivariate) = 1.20 (0.88 - 1.65), p < 0.243].
Dupont et al., 2012^8^	Risk of death from any cause and non-fatal cardiovascular event of patients in the fourth quartile was higher than in patients in the first quartile (p = 0.002).
Gao et al., 2011^21^	Patients who developed fatal or non-fatal cardiovascular events showed higher levels of cystatin C compared to patients who did not develop these events (1.63 ± 0.81 mg/L x 0.91 ± 0.27 mg/L, p = 0.001)Risk of fatal or non-fatal cardiovascular events in patients with cystatin C levels> 0,9 mg/L was higher than in patients with cystatin levels < 0.9 mg/L [(univariate) HR = 3.58 (2.61 - 4.82), p = 0.033; HR (multivariate) = 7.10 (3.36 - 23.75), p = 0,006].
Keller et al., 2009^17^	Patients with cardiovascular death had higher levels of cystatin C than patients without cardiovascular death [0.94 (0.79 - 1.08 x 0.79 (0.70 - 0.90), p < 0.001].Risk of cardiovascular death of patients in the fourth quartile was higher than in patients in the other quartiles [OD (univariate) = 3.87 (2.33-6.42), p < 0.001; OD (multivariate) = 1.86 (0.90-3.81), p = 0.09].
Gao et al., 2009^22^	Patients with AMI and unstable angina had higher levels of cystatin C than the control group (2873.55 ± 1148.48 ng/mL x 1509.99 ± 408.65 ng/mL, p < 0.01 and 2013.83 ± 633.85 ng/mL x 1509.99 ± 408.65 ng/mL, p < 0.05, respectively).Patients with AMI and unstable angina had higher levels of cystatin C than the patients with stable angina (2873.55 ± 1148.48 ng/mL x 1348.41 ± 369.62 ng/mL, p < 0.01 and 2013.83 ± 633.85 ng/mL x 1348.41 ± 369.62 ng/mL, p < 0.01, respectively).Patients with AMI had higher levels of cystatin C than the patients with stable angina (2873.55 ± 1148.48 ng/mL x 2013.83 ± 633.85 ng/mL, p < 0.05).Patients who developed fatal or non-fatal cardiovascular events showed higher levels of cystatin C compared to patients who did not develop these events (2356,73 ± 897,64 ng/L x 1469.51 ± 574.83 ng/L, p = 0.006)
Alehagen et al., 2009^20^	Risk of cardiovascular death of fourth quartile patients was higher than that of first quartile patients [HR (univariate analysis) = 3.61 (1.81 - 7.14)].
Acuna et al., 2009^16^	The proportion of patients with cystatin C levels > 0.95 mg/L who had cardiovascular death was higher than that of patients with cystatin C levels ≤ 0.95 mg/L [16 (27.1%) x 6 (7.8%), p = 0.01].The proportion of patients with cystatin C levels> 0.95 mg/L who develop HF was higher than that of patients with cystatin C levels ≤ 0.95 mg/L [22 (40.7%) x 6 (7.5%), p = 0.01].
Koenig et al., 2007^24^	Each increase of 0.18 mg/L cystatin C was associated with an increased risk of cardiovascular death [OD = 1.42 (1.30 -1.54)], death from any cause [OD = 1.33 1.25-1.40)], HF [OD = 1.28 (1.17-1.40)], stroke [OD = 1.22 (1.08-1.38)] and AMI [OD = 1.20 (1.06-1.36)].Patients with high levels of cystatin C had more adverse events than those with reduced levels of cystatin C (p < 0.001).
Ix et al., 2007^23^	Risk of death from any cause of fourth quartile patients was higher than that of first quartile patients [HR (univariate) = 5,7 (3,1 - 10,5), p < 0.001; HR (multivariate) = 3,6 (1,8 - 7,0), p < 0.001].Risk of cardiovascular events of fourth quartile patients was higher than that of first quartile patients [HR (univariate) = 3.8 (2.1 - 6.9), p < 0.001; HR (multivariate) = 2.0 (1.0 - 3.8), p < 0.04].Risk of CHF in patients in the fourth quartile was higher than in patients in the first quartile [HR (univariate) = 6.1 (2.5 - 14.5), p = 0.001; HR (multivariate) = 2.6 (1.0 - 6.9), p = 0.05].

CAD: Coronary artery disease; AMI: Acute Myocardial Infarction; GFR: Glomerular filtration rate; HR: Hazard Ratio.

### Studies results

Among the included studies, two (16.66%)^16,19^ analyzed the difference between the proportion of patients with high levels of cystatin C who developed fatal or non-fatal cardiovascular events,^19^ cardiovascular death,^16^ and CHF^16^ compared with the proportion of patients with reduced levels of Cystatin C that developed these events, and all of them found a significant difference. A study (8.33%)^24^ further observed that patients with high levels of cystatin C had more adverse cardiovascular events than those with reduced levels of cystatin C. The difference between cystatin C levels in patients who developed fatal or non-fatal cardiovascular events, and those who did not develop these events was evaluated by four studies (33.33%),^7,17,19,21^ and all found significantly higher levels of cystatin C in the group of patients who developed the events. A study (8.33%)^18^ also found that cystatin C levels in patients with CAD were higher than in the control group and another study (8.33%)^22^ observed that cystatin C levels in patients with AMI were higher than in patients with unstable angina, stable angina, and control group, and that cystatin C levels in patients with unstable angina were higher than in those with stable angina and control group. Another study (8.33%)^7^ found a higher survival rate in patients with lower levels of cystatin C.

The risk of developing adverse outcomes was assessed by eight studies (66.66%)^15,17-21,23,24^ calculating the hazard ratio. Among these, two studies (22,22%)^19,21^ found an increased risk of fatal or non-fatal cardiovascular events in patients with higher levels of cystatin C; one study (11.11%)^18^ observed a higher risk of death from any cause and non-fatal cardiovascular events; another study found an increased risk of cardiovascular death and death from any cause; two studies (22.22%)^17,20^ found an increased risk of cardiovascular death; one study (11.11%)^23^ found an increased risk of death from any cause, cardiovascular events and CHF; and one study (11.11%)^15^ still observed a higher risk of left ventricular hypertrophy. Finally, one study^24^ found that each increase of 0.18 mg/L of cystatin C was associated with an increased risk of cardiovascular death, death from any cause, HF, stroke and AMI. The multivariate regression analysis was performed by six (50%)^15,17-19,21,23^ of these studies, with the risk of developing evaluated adverse outcomes remaining significant after the performance of this analysis in four of these studies.^18,19,21,23^

### Methodological quality

The results of the evaluation of the methodological quality of the studies included in this review are shown in Table 5, and the detailed description of the criteria used for the distribution of the stars is presented in the legend. After the quality analysis, a study (8.33%)^22^ was found to have good methodological quality and 11 studies (91.66%) had excellent methodological quality.

**Table 5:** Evaluation of study quality according to Newcastle-Ottawa Scale

Author/Year	Selection 1 2 3 4				Comparability 5	Outcomes 6 7 8			Total score
Sai *et al*., 2016^19^	*	*	*	-	**	*	*	*	8
Bansal et al., 2016^15^	*	*	*	-	**	*	*	*	8
Abid et al., 2016^7^	*	*	*	-	*	*	*	*	7
Woitas *et al*., 2013^18^	*	*	*	-	**	*	*	*	8
Dupont et al., 2012^8^	*	*	*	-	*	*	*	*	7
Gao et al., 2011^21^	*	*	-	-	**	*	*	*	7
Keller *et al*., 2009^17^	*	*	*	-	**	*	*	*	8
Gao et al., 2009^22^	*	*	-	-	*	*	-	*	5
Alehagen et al., 2009^20^	*	*	*	-	*	*	*	*	7
Acuna et al., 2009^16^	*	*	*	-	*	*	*	*	7
Koenig *et al*., 2007^24^	*	*	-	*	*	*	*	*	7
Ix et al., 2007^23^	*	*	*	-	**	*	*	*	9

1 - Representativeness of the exposed cohort: all the studies received one star, because the exposed cohort was a little representative of the average in the community; 2 - Selection of the unexposed cohort: all studies received one star, because the unexposed cohort was obtained in the same community of the exposed cohort; 3- Determination of exposure: only studies that dosed cystatin C using the immunonephelometry or immunoturbidimetry methods received a star; 4 - Demonstration that the outcome of interest was not present at the beginning of the study: studies in which patients did not present any cardiovascular disease at the beginning of the study received one star; 5 - Cohort comparability based on design and analysis: studies that performed multivariate regression analysis of Cox proportional hazards and defined normal renal function as GFR > 60 mL/min/1.73 m^2^ received 2 stars. Studies that only defined normal renal function as GFR > 60 mL/min/1.73 m^2^ but did not perform multivariate regression analysis of Cox proportional hazards received 1 star. 6 - Determination of outcome: all studies received one star, because the evaluation of the outcome was performed by the physicians independently; 7 - Adequate follow-up period for the occurrence of outcome (s): studies in which patients were followed for at least six months received one star, and studies in which patients were followed for less than six months did not receive a star; 8 - Adequacy of the follow-up period of the cohort: studies in which at least 90% of the patients were followed to the end or who did not comment if there were significant loss of patients during follow-up received one star.

### Meta-analysis

**Figure 2:**
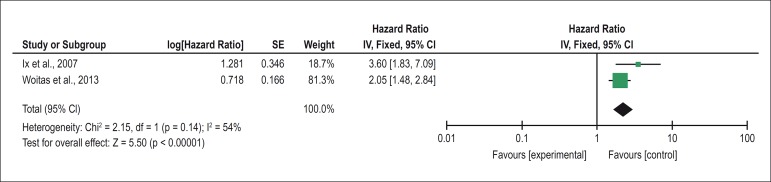
Metanalysis of studies evaluating the association between high levels of cystatin C and the risk of mortality from any cause through the comparison between the fourth and first quartiles of cystatin C.

Only two studies evaluated the outcome of all-cause mortality, compared the fourth quartile of cystatin C with the first quartile, and performed a multivariate regression analysis of Cox proportional hazards and were therefore included in the meta-analysis, the result of which is shown in Figure 2. Homogeneity was observed among the studies (I^2^ = 53,423 and p = 0,14); therefore, the fixed-effect model was used to calculate the hazard ratio. The result of the meta-analysis [HR = 2.28 (1.70 - 3.05), p < 0.001] indicates that there is a significant association between high levels of cystatin C and the risk of all-cause mortality in individuals with normal renal function. A symmetric distribution of the articles included in the meta-analysis was observed in the *funnel plot*, indicating that there is no publication bias.

## Discussion

The present study aimed to evaluate the association between high levels of cystatin C and the risk of cardiovascular events or mortality in subjects with normal renal function through a systematic review of the scientific literature and meta-analysis.

The difference between the proportion of patients with high levels of cystatin C who developed cardiovascular events or mortality, compared with the proportion of patients with reduced levels of Cystatin C that developed these events was evaluated by two studies and both of them found a significant difference. The difference between cystatin C levels in patients who developed fatal or non-fatal cardiovascular events and those who did not develop these events was assessed by four studies (33.3%) and all found significantly higher levels of cystatin C in the group of patients who had the events. The risk of developing adverse outcomes was assessed by eight studies (66.66%) calculating the hazard ratio. Among these, six studies found an increased risk of cardiovascular events or mortality. The multivariate regression analysis was performed by six (50%) of these studies, with the risk of developing the adverse outcomes remaining significant after the performance of this analysis in four of these studies.

The meta-analysis also demonstrated that there is a significant association between high levels of cystatin C and the risk of all-cause mortality. Thus, the results presented by the studies included in this systematic review and meta-analysis indicate that there is a significant association between high levels of cystatin C and the development of cardiovascular events or mortality in subjects with normal renal function assessed by serum creatinine-based GFR.

A possible mechanism for the association between high levels of cystatin C and the development of cardiovascular events is related to the atherogenic process. The development of lesions in the arteries endothelium results in the accumulation of cholesterol in the artery wall, and in the development of the atherosclerotic plaque.^25^ It has been suggested that lysosomal cathepsins, whose production is stimulated by inflammatory cytokines, may contribute to the degradation of the atherosclerotic plaque. As cystatin C is able to inhibit lysosomal cathepsins, it is possible to suggest that elevated levels of cystatin C may contribute to non-degradation of atherosclerotic plaque, resulting in increased risk of cardiovascular events.^26,27^

Another possible mechanism is related to the fact that cystatin C presents a greater sensitivity for the detection of the initial stages of renal dysfunction than serum creatinine or creatinine-based GFR.^28,29^ Several authors have already demonstrated that renal dysfunction is associated with an increased risk of cardiovascular events.^30,31^ Thus, it is possible to suggest that patients who have normal renal function assessed by GFR based on creatinine or serum creatinine but who have high levels of cystatin C may present with renal dysfunction at an earlier stage, which could be associated with an increased risk of cardiovascular events.

Although cystatin C is a more sensitive marker for detecting the early stages of CKD than creatinine, especially in groups at risk for CKD, such as patients with diabetes mellitus and renal transplant recipients, it has some limitations.^32,33^ High doses of glucocorticoids and hyperthyroidism may result in increased serum levels of cystatin C, whereas hypothyroidism may result in a decrease.^34^ Some factors, such as age, male gender, body weight, smoking, C-reactive protein, cancer, inflammatory processes and steroid therapy may also influence serum levels of cystatin C, limiting its assessment in clinical practice.^35^

Renal weight and volume decrease gradually between the ages of 30 and 90 years, resulting in a natural decline of renal function with increasing age.^36^ Thus, elderly patients have a lower GFR, which may be associated with higher levels of cystatin C and an increased risk of cardiovascular events.^28^ As most of the studies that performed the multivariate regression analysis [66.66% (n = 4)]^15,17,18,23^ included age in this analysis, and nonetheless found a significant association between high levels of cystatin C and the development of adverse outcomes, it is possible to conclude that this association is age-independent. It should be noted that the two studies^20,25^ that were included in the meta-analysis are among these studies that included age in the multivariate regression analysis, indicating that the association between high levels of cystatin C and any cause-related mortality observed in meta-analysis is age-independent.

All selected studies have described the renal function of patients as being normal. The estimated GFR calculated by the MDRD formula, greater than 60 mL/min/1.73 m^2^, was used as a criterion for normal renal function in 66.67% of the studies, and 8.33% used serum creatinine levels below 115 µmol/L. The estimated GFR is a better marker for renal function evaluation than serum creatinine, because it undergoes interference of muscle mass, gender, age, physical activity and diet. Moreover, unlike GFR, serum creatinine is not able to detect the presence of chronic renal disease early because its levels increase only when renal disease is already at an advanced stage.^31^ The inclusion of individuals with estimated GFR greater than 60 mL/min/1.73 m^2^ by most studies, including studies of the meta-analysis, supports the information that the association between high levels of cystatin C and the risk of cardiovascular events or mortality is not dependent on the renal function of the patient evaluated by creatinine-based estimated GFR, which is a marker that has good sensitivity for the detection of renal dysfunction in the early stages.

Immunonephelometry and immunoturbidimetry were the most commonly used methods [75% (n = 9)] for the laboratory dosage of cystatin C and were even used by the studies included in the meta-analysis. These methods have good precision, specificity, adequate time to result, and minimum amount of sample required, being the methods of choice for cystatin C^37,38^ dosage. Therefore, the use of these methods by most of the studies included in the systematic review brings greater reliability to the results.

The sample size of the studies ranged from 127 to 4,663 individuals, with most of them having more than 400 individuals [58.33% (n = 7)].^8,15,17,18,20,23,24^ The study^7^ that obtained the smallest sample size still included more than 100 individuals, which can be considered a significant number if the follow-up is performed for an adequate time.^39^ It should be noted that this study found a significant difference between patients who developed fatal or non-fatal cardiovascular events and those who did not develop these events.

This systematic review had some limitations, such as the population studied, which varied widely among the studies. Only one study^24^ included healthy elderly subjects, while the population of the other studies consisted of patients at risk for cardiovascular events,^15^ with STEMI and NSTEMI,^7,16^ with stable CAD,^17,18^ SCA,^17^ patients undergoing percutaneous coronary intervention,^19^ with CHF,^20,21^ with CHF who underwent coronary angiography,^8^ with stable angina and AMI,^22^ and with a history of AMI that had angiographic evidence of stenosis greater than 50%.^23^ This variation may lead to bias in the results, because cardiovascular impairment varied among the populations at the beginning of the studies, which may influence cystatin C levels, since patients with CHF or AMI could present higher levels of cystatin C at the beginning of the study if compared to patients who only present risk of cardiovascular events.^23^ Since most studies evaluated a population at risk of cardiovascular events or who already have some degree of cardiovascular impairment, it is possible to suggest that cystatin C is an interesting marker for assessing the risk of cardiovascular events or mortality in these population groups and may complement the currently available markers.

In addition to the variation of the study population, follow-up time, patient classification, and outcomes also varied widely across studies. The follow-up time ranged from six months to ten years, and three studies (25%)^7,16,22^ followed the patients for less than 15 months and four studies (33.33%)^15,18,20,24^ have followed for more than nine years. The prevalent time of follow-up of the studies was three to six years [41.67% (n = 5)].^8,17,19,21,23^ The follow-up time should be adequate for the outcome to be observed, and should be greater for the detection of mortality than for cardiovascular events. The study^22^ with shorter follow-up (6 months) found higher levels of cystatin C among patients who developed fatal and non-fatal cardiovascular events compared to patients who did not develop these outcomes, indicating that even shorter follow-up time was sufficient for the detection of both outcomes and for the observation of a significant association with Cystatin C levels. Both studies included in the meta-analysis assessed the outcome for all-cause mortality. One of them followed the patients for three years and the other for ten years, with these times being adequate for the evaluation of the outcome.

Patients classification to carry out the statistical analysis also varied considerably among the studies. Only five studies (41.66%),^8,17,18,20,23^ including the studies of the meta-analysis, classified patients according to quartiles of cystatin C, which is the best classification to establish a cutoff point above which the risk of developing cardiovascular events or mortality would be higher.

Despite these study limitations, of the articles selected in this systematic review, 11 have excellent methodological quality and only one has good quality.

## Conclusion

The systematic review has shown that there is a significant association between high levels of cystatin C and the risk of cardiovascular events or mortality in subjects with normal renal function. The meta-analysis also demonstrated that there is a significant association between high levels of cystatin C and the risk of all-cause mortality. As individuals included in the studies had normal renal function, it is possible to conclude that the association between high levels of cystatin C and the risk of cardiovascular events or mortality does not depend on the presence of renal dysfunction assessed by serum creatinine-based GFR. Therefore, cystatin C is a very interesting marker to assess the risk of cardiovascular events or mortality, especially in populations at risk of cardiovascular events or that already have some degree of cardiovascular impairment, and can complement the currently available markers.
